# Dynamic Supraglottic Airway Collapse Diagnosed Using Airway Point-of-care Ultrasound: A Case Report

**DOI:** 10.5811/cpcem.61946

**Published:** 2026-06-29

**Authors:** Arihant Jain, Anas Mohammed Muthanikkatt, S Manu Ayyan

**Affiliations:** * All India Institute of Medical Sciences, Department of Emergency Medicine, New Delhi, India; † Jawaharlal Institute of Postgraduate Medical Education and Research, Department of Emergency Medicine, Pondicherry, India

**Keywords:** acquired laryngomalacia, dynamic airway collapse, airway pocus, emergency medicine, adolescent stridor, case report

## Abstract

**Introduction:**

Acquired idiopathic laryngomalacia causing dynamic airway collapse is rare in adolescents and may be overlooked because it mimics more common causes of acute dyspnoea and stridor in the emergency department (ED). Airway point-of-care ultrasound (POCUS) provides a rapid, non-invasive means to visualize dynamic supraglottic obstruction when laryngoscopy is not immediately feasible or tolerated.

**Case Report:**

A 13-year-old boy presented with sudden-onset respiratory distress and dyspnoea that worsened on lying flat and improved when sitting upright, without history of fever, trauma, allergy, or foreign body aspiration, and with similar prior self-limiting episodes. He was anxious with nasal flaring, subcostal retractions, tachypnoea, tachycardia, distended neck veins, and a squeaky tracheal inspiratory sound, yet he maintained normal oxygen saturation with equal air entry on auscultation. Airway POCUS showed abnormal downward displacement of the epiglottis with every distressful inspiration, indicating dynamic airway collapse, and non-contrast computed tomography of the neck demonstrated a heart-shaped, mid-epiglottis deformity consistent with acquired idiopathic laryngomalacia. The patient was given supportive treatment to reduce mucosal oedema, resulting in gradual symptom resolution, and after two days of observation and otorhinolaryngology evaluation he was discharged in stable condition.

**Conclusion:**

Acquired idiopathic laryngomalacia should be considered in adolescents with unexplained positional respiratory distress and discordant findings of significant work of breathing but preserved oxygenation. Airway point-of-care ultrasound can delineate real-time dynamic laryngeal abnormalities at the bedside, thereby facilitating early diagnosis and appropriate management of dynamic airway collapse in the emergency department.

## INTRODUCTION

Acquired idiopathic laryngomalacia is an uncommon cause of upper airway obstruction in adolescents, in contrast to the predominantly congenital presentation seen in infancy.^1^–^6^ Its symptoms often overlap with more prevalent cardiorespiratory or functional disorders, leading to under-recognition of subtle dynamic supraglottic collapse in the emergency department (ED).^3^–^5^,^7^ In many such cases, the apparent “marginal” airway is managed with sedative medications for agitation or for planned laryngoscopy, which can precipitate complete airway compromise and unnecessary intubation when dynamic collapse worsens under sedation. 3,4,8

These diagnostic challenges are compounded by the limitations of conventional laryngoscopy, which is invasive, requires a cooperative or sedated patient, and may be difficult to perform safely in those with labile airway physiology.^3^,^4^,^8^ Airway point-of-care ultrasound (POCUS) offers a rapid, non-invasive, real-time modality to visualise dynamic laryngeal motion at the bedside, allowing clinicians to identify occult supraglottic collapse before proceeding to potentially hazardous sedation or intubation.^9^–^12^ This case highlights how focused airway POCUS can uncover acquired idiopathic laryngomalacia in an adolescent whose symptoms could easily have been misattributed, thereby supporting more targeted, conservative management and avoiding escalation to unnecessary invasive airway procedures.^1^–^5^,^7^,^9^,^10^

## CASE REPORT

A 13-year-old boy presented to the ED with a two-day history of acute onset breathing difficulty that worsened with exertion and when lying supine and improved on sitting upright and calming down. His caregivers reported intermittent abnormal inspiratory sounds that were most prominent during episodes of distress and on deep inspiration, occasionally accompanied by a runny nose but without other allergic manifestations. There was no history of fever, trauma, easy fatigability, leg swelling, palpitations, syncope, or foreign body aspiration; his antenatal, perinatal, and developmental history were unremarkable. He had experienced three similar prior episodes over the preceding two years, including one in the prior two months, which had been attributed to functional or anxiety-related symptoms and often followed upper respiratory tract infections or mild allergy.

On arrival, he was conscious, oriented, and appeared in respiratory distress with nasal flaring and visible abnormal laryngeal movement (Video 1). His vital signs were as follows: blood pressure, 120/80 millimetres of mercury; heart rate, 100 beats per minute; respiratory rate, 40 breaths per minute; oxygen saturation, 99% on room air; capillary refill time < 3 seconds, afebrile temperature; and random blood glucose 120 milligrams per decilitre (mg/dL) (reference range: 70–140 mg/dL). Arterial blood gas analysis demonstrated respiratory alkalosis, and the electrocardiogram revealed sinus tachycardia. Chest auscultation documented equal air entry bilaterally despite increased work of breathing and a squeaky tracheal inspiratory sound. Cardiovascular, abdominal, and neurological examinations were within normal limits.

Given the discordance between marked respiratory effort and preserved oxygenation, an upper airway or otorhinolaryngologic cause was considered, with a working differential that included infectious laryngotracheitis, foreign body, traumatic injury, structural lesions, allergic or angioedema-related swelling, external compression, and functional causes. Fiber-optic bronchoscopy was contemplated but deferred because of its invasive nature, the need for sedation, and the patient’s acute distress. Airway POCUS was selected as the next diagnostic step.

*CPC-EM Capsule*

What do we already know about this clinical entity?

*Acquired idiopathic laryngomalacia is a rare cause of dynamic supraglottic collapse in adolescents.​*

What makes this presentation of disease reportable?

*Use of airway point-of-care ultrasound (POCUS) avoided escalation to invasive airway interventions.​*

What is the major learning point?

*Focused airway POCUS can visualize realtime supraglottic dynamics, revealing occult laryngomalacia.​*

How might this improve emergency medicine practice?

*Incorporating airway POCUS for atypical stridor and positional respiratory distress may expedite diagnosis of dynamic airway collapse and guide safer airway strategies.*

Airway POCUS was performed with a high-frequency linear transducer placed in the transverse plane at the level of the supraglottis and glottis. At the epiglottic level, real-time imaging revealed abnormal downward displacement and prolapse of the anterior portion of the epiglottis into the airway lumen during inspiration, resulting in dynamic narrowing (Video 2). At the level of the vocal cords, we noted symmetric inward medial movement of the cords with each inspiratory effort, making vocal cord dysfunction or palsy an unlikely cause of the symptoms.

Non-contrast computed tomography of the neck, obtained during inspiratory effort in the supine position, demonstrated a characteristic heart-shaped deformity of the mid-epiglottis at the level of the piriform sinuses, without evidence of mass, foreign body, or external compression (Image).

In the ED the patient received nebulized adrenaline (0.5 mL per kilogram of 1:1000 solution diluted to total of 5 mL with normal saline, maximum dose of 5 mL) and intravenous hydrocortisone (1 mg/kg) targeting presumed supraglottic oedema, along with close airway and cardiorespiratory monitoring. His work of breathing and abnormal inspiratory sounds improved after repeated nebulisation treatment, and serial assessments demonstrated stable oxygenation and haemodynamics without progression to overt upper airway obstruction. He was admitted for observation under the otorhinolaryngology (ENT) team, managed conservatively, and did not require intubation or surgical intervention. Over the next few days, his exertional flareups settled and his overall symptoms improved. Telelaryngoscopy showed supraglottic oedema of uncertain cause that continued to respond to conservative treatment, and once the oedema had resolved and there were no further exertional symptoms, he was discharged home after his parents were counselled about warning signs and an ENT outpatient review was arranged.

## DISCUSSION

Acquired laryngomalacia is classically described as a dynamic inspiratory supraglottic collapse in older children and adults, most often involving redundant epiglottic tissue, aryepiglottic folds, or arytenoids prolapsing into the laryngeal inlet.^1^,^3^–^5^ Although increasingly reported in case series and reviews, it remains rare and easily missed outside ENT settings.^3^,^4^ In contrast to the predominantly congenital form in infants, which presents with chronic inspiratory noise and feeding difficulty, late-onset laryngomalacia tends to manifest with exertional or positional dyspnoea, stridor, cough, and voice changes, and may follow neurologic disease, airway surgery/iatrogenic, age-related tissue laxity, exercise-induced collapse, and idiopathic cases.^1^,^3^–^5^,^7^ Adult and late-onset cases have also been implicated in post-extubation stridor and extubation failure when dynamic supraglottic collapse is not anticipated.^3^,^8^

In this adolescent patient, episodic respiratory distress with positional worsening, preserved oxygen saturation, and largely unremarkable chest findings initially resembled more common cardiorespiratory or functional disorders. Similar diagnostic delay is described in adults with laryngomalacia or expiratory central airway collapse, where symptoms are often attributed to asthma, chronic obstructive pulmonary disease, or anxiety until dynamic imaging is pursued.^3^,^4^,^13^,^14^ Expiratory central airway collapse encompasses trachea-bronchomalacia and excessive dynamic airway collapse, characterized by expiratory collapse of the trachea and main bronchi, in contrast to the inspiratory supraglottic collapse observed in laryngomalacia; both conditions, however, rely on dynamic visualization for definitive diagnosis and are increasingly recognized as underappreciated causes of “unexplained” dyspnoea, cough, and extubation failure.^13^–^15^

Awake or flexible laryngoscopy performed during an active episode is considered the diagnostic standard for adult laryngomalacia, whereas dynamic bronchoscopy and dynamic computed tomography are central to evaluating expiratory central airway collapse.^3^,^4^,^13^–^15^ These modalities provide detailed structural assessment but require specialized equipment, patient cooperation, and, frequently, some degree of sedation. In emergency practice, this creates a vulnerable window in which a marginal airway appears manageable while the patient is awake yet may decompensate after sedatives are administered for diagnostic maneuvers. Case reports of acquired laryngomalacia describe scenarios where unrecognized supraglottic collapse contributed to post-extubation stridor, failed extubation, or rapid progression to invasive airway interventions once protective upper airway tone was lost.^1^,^4^,^8^

The case we present here illustrates how airway POCUS can help bridge this gap in the ED. Dynamic sonography at the supraglottic level demonstrated abnormal inspiratory prolapse of the anterior epiglottis into the airway lumen, while imaging at the level of the vocal cords showed symmetric inward medial movement with each inspiratory effort, making vocal cord paralysis or paradoxical vocal cord motion unlikely as the primary mechanism (Video 2). These ultrasound findings were corroborated by non-contrast computed tomography (CT) of the neck in the supine position during inspiration, which revealed a heart-shaped deformity of the mid-epiglottis without mass, foreign body, or external compression (Image).

Ultrasound is already well established for several aspects of airway management, including prediction of difficult laryngoscopy, confirmation of endotracheal tube placement, assessment of laryngeal oedema, and localization of the cricothyroid membrane.^11^,^12^ Contemporary reviews, however, note that specific, validated sonographic criteria for diagnosing laryngomalacia or expiratory central airway collapse have not yet been defined and that these entities remain primarily endoscopic and CT diagnoses.^3^,^13^–^15^ Pediatric data demonstrate that laryngeal ultrasound can predict severe laryngomalacia and aid in the evaluation of stridor, supporting the concept that dynamic supraglottic collapse is sonographically accessible, even though routine protocols for adolescents and adults are lacking.^9^,^10^ In this case, airway ultrasound did not replace formal ENT evaluation but provided an early, non-invasive view of supraglottic dynamics that guided conservative, airway-preserving management while avoiding immediate sedated laryngoscopy or bronchoscopy.

Management strategies for acquired laryngomalacia span a continuum from observation and respiratory retraining or speech therapy to supraglottoplasty, epiglottopexy, partial epiglottidectomy, arytenoidectomy, or tracheostomy in severe or neurologically compromised patients.^1^,^3^–^5^,^7^ Surgical intervention is reported in most symptomatic adults, with substantial—but not universal—symptom relief, whereas management of expiratory central airway collapse often involves optimization of underlying lung disease, positive airway pressure, airway clearance measures, short-term stent trials, and, in selected cases, trachea-bronchoplasty.^13^–^15^ In contrast, the adolescent patient described here improved with targeted medical therapy (adrenaline nebulization and systemic corticosteroids for presumed supraglottic oedema), close airway monitoring, and ENT input, illustrating that dynamic supraglottic collapse in younger patients may be reversible without surgery when promptly recognized and linked to potentially inflammatory precipitants.^2^,^9^,^10^

Several practical implications for emergency clinicians emerge from this case. First, adolescents and adults presenting with positional or exertional respiratory distress, discordant clinical findings and oxygenation, and atypical stridor warrant consideration of dynamic supraglottic pathology, including acquired laryngomalacia, alongside more familiar lower airway diagnoses.^1^–^5^ Second, routine sedation of such patients for laryngoscopy or intubation can unmask catastrophic airway collapse if dynamic structural instability is present; whenever feasible, dynamic assessment while the patient is awake and maintaining spontaneous ventilation should be prioritized.^3^,^4^,^8^,^13^ Third, focused airway POCUS, although not yet a validated stand-alone diagnostic test for laryngomalacia, can depict real-time supraglottic motion, help distinguish structural collapse from vocal cord dysfunction, and support risk-stratified decisions about proceeding to sedated endoscopy or invasive airway interventions.^9^–^12^ Further research is needed to standardize airway POCUS views, define diagnostic thresholds, and clarify how ultrasound can be integrated with laryngoscopy and CT in the evaluation of dynamic airway collapse across the age spectrum.^3^,^9^–^15^

## CONCLUSION

Acquired idiopathic laryngomalacia should be considered in adolescents with positional or exertional respiratory distress and discordant findings of significant work of breathing but preserved oxygenation, as unrecognized dynamic supraglottic collapse may deteriorate precipitously with sedation or intubation. Focused airway point-of-care ultrasound can complement laryngoscopy by providing real-time visualization of supraglottic dynamics at the bedside, enabling earlier recognition, guiding safer airway strategies and, in selected patients, allowing conservative, airway-preserving management.

## Supplementary Information

Video 1 Head and neck examination demonstrating nasal flaring and abnormal vertical movement of the laryngeal framework with each inspiratory effort in a young patient who presented with sudden-onset respiratory distress and dyspnoea.

Video 2 Airway point-of-care ultrasound at the epiglottic level demonstrating inward prolapse of the mid-epiglottis (arrow) into the airway lumen with each inspiratory effort in an adolescent young patient who presented with sudden-onset respiratory distress and dyspnoea.

## Figures and Tables

**Image:**
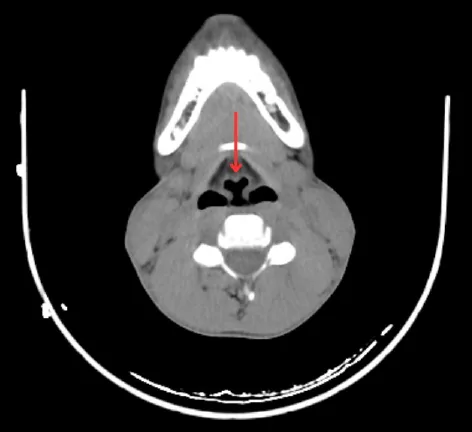
Non-contrast computed tomography of the neck at the level of the epiglottis obtained during inspiration, demonstrating abnormal inward prolapse of the mid-epiglottis (arrow) creating a heart-shaped airway configuration in an adolescent young patient who presented with sudden-onset respiratory distress and dyspnoea.

## References

[1] Kawamoto A, Katori Y, Honkura Y (2013). Acquired idiopathic laryngomalacia treated by laser supraglottic laryngoplasty. Tohoku J Exp Med.

[2] Kim M, Lee S, Shin M (2024). Acquired idiopathic laryngomalacia in a 12-year-old adolescent: a case report. Allergy Asthma Respir Dis.

[3] Mills J, Monaghan N, Nguyen S (2024). Adult laryngomalacia: a scoping review. Otolaryngol Head Neck Surg.

[4] Ferri G, Prakash Y, Levi J (2020). Differential diagnosis and management of adult-onset laryngomalacia. Am J Otolaryngol.

[5] Hey S, Oozeer N, Robertson S (2014). Adult-onset laryngomalacia: case reports and review of management. Eur Arch Otorhinolaryngol.

[6] Siou GS, Jeannon JP, Stafford FW (2002). Acquired idiopathic laryngomalacia treated by laser aryepiglottoplasty. J Laryngol Otol.

[7] Suresh I, Challa S, Gattavadi SN (2021). Phagosyncope: an unusual presentation of airway malfunction due to acquired laryngomalacia in an elderly Indian male. Int J Otorhinolaryngol Head Neck Surg.

[8] Mizunoya K, Onodera K, Takahashi Y (2023). Acquired laryngomalacia as a cause of post-extubation stridor and extubation failure following craniotomy: a case report. JA Clin Rep.

[9] Duantaweesook A, Vathanophas V, Ungkanont K (2025). Laryngeal ultrasound for the prediction of severe laryngomalacia. PLOS One.

[10] Kaur T, Baijal N, Jana M (2024). Ultrasound in causes of stridor in children. J Ultrasound Med.

[11] Lin J, Bellinger R, Shedd A (2023). Point-of-care ultrasound in airway evaluation and management: a comprehensive review. Diagnostics.

[12] Khorsand S, Chin J, Rice J (2023). Role of point-of-care ultrasound in emergency airway management outside the operating room. Anesth Analg.

[13] Aslam A, De Luis Cardenas J, Morrison R (2022). Tracheobronchomalacia and excessive dynamic airway collapse: current concepts and future directions. Radiographics.

[14] Kheir F, Majid A (2018). Tracheobronchomalacia and excessive dynamic airway collapse: medical and surgical treatment. Semin Respir Crit Care Med.

[15] Pu C, Keyes C, Majid A (2025). Expiratory central airway collapse: a comprehensive narrative review. Clin Chest Med.

